# A Framework for Implementing Metaheuristic Algorithms Using Intercellular Communication

**DOI:** 10.3389/fbioe.2021.660148

**Published:** 2021-05-10

**Authors:** Yerko Ortiz, Javier Carrión, Rafael Lahoz-Beltrá, Martín Gutiérrez

**Affiliations:** ^1^School of Informatics and Telecommunications, Faculty of Engineering and Sciences, Diego Portales University, Santiago, Chile; ^2^Department of Biodiversity, Ecology and Evolution (Biomathematics), Faculty of Biological Sciences, Complutense University of Madrid, Madrid, Spain

**Keywords:** agent based model, synthetic biology, bioinspired algorithms, cell-cell communication, metaheuristics, framework, *gro*

## Abstract

Metaheuristics (MH) are Artificial Intelligence procedures that frequently rely on evolution. MH approximate difficult problem solutions, but are computationally costly as they explore large solution spaces. This work pursues to lay the foundations of general mappings for implementing MH using Synthetic Biology constructs in cell colonies. Two advantages of this approach are: harnessing large scale parallelism capability of cell colonies and, using existing cell processes to implement basic dynamics defined in computational versions. We propose a framework that maps MH elements to synthetic circuits in growing cell colonies to replicate MH behavior in cell colonies. Cell-cell communication mechanisms such as quorum sensing (QS), bacterial conjugation, and environmental signals map to evolution operators in MH techniques to adapt to growing colonies. As a proof-of-concept, we implemented the workflow associated to the framework: automated MH simulation generators for the *gro* simulator and two classes of algorithms (Simple Genetic Algorithms and Simulated Annealing) encoded as synthetic circuits. Implementation tests show that synthetic counterparts mimicking MH are automatically produced, but also that cell colony parallelism speeds up the execution in terms of generations. Furthermore, we show an example of how our framework is extended by implementing a different computational model: The Cellular Automaton.

## Introduction

Evolution is a trademark process involved in all living organisms. It is the process that drives organism adaptation to better survive and thrive in their surrounding environment. This process occurs at a genotypic level involving mainly genetic recombination and mutations of DNA material which translates into potential changes at a phenotypic level of the organism. Appropriate and useful organism phenotypical traits are selected and passed onto newly generated offspring, who in turn recombine and mutate their genetic material. It is in following this cycle that organisms evolve and develop new useful phenotypical traits, while other traits are lost (Darwin and Costa, 2009; Freeman and Herron, 2015; Futuyma and Kirkpatrick, 2017; Losos, 2017). The transformation of the individual organisms within populations can be seen as a trial-and-error process in which each variation is tested for adaptation to the environment conditions. Genetic diversity produced by evolution is studied and used as inspiration in computational methods such as Evolutionary Algorithms (EAs) (Bäck, 1996; De Jong, 2016). These algorithms are generally used for approximating solutions to optimization problems and rely on a pool of potential solutions that evolve over time.

Since Evolution is a standard occurring process in all living organisms, it is natural to relate EAs to them. Specifically, microbiology experiments and Synthetic Biology constructs can be used as a platform for carrying out the execution of said algorithms, as cell colonies offer immense processing power coming from each individual cell, and large counts of these entities function simultaneously. Also, natural processes involved in cell operation, such as recombination or mutation originally inspire EAs. For instance, synthetic bacteria have been a source of inspiration for the design of EAs illustrating its utility solving simple instances of optimization problems such as function optimization, 0/1 knapsack problem and Hamiltonian path problem (Gargantilla Becerra et al., 2021). Therefore, using these processes in the design and implementation in a cell colony mimics their computational counterpart. Native cell processes such as growth, mutation and intercell communication are components that can be mapped to tasks pertaining to computational EAs, greatly easing the translation. The described relationship has already been addressed by Directed Evolution (Arnold, 1998, 2018) and its variants (English et al., 2019; Wu et al., 2019; Yang K. K. et al., 2019; Morrison et al., 2020). However, the control level of Directed Evolution is not as specific as the one reached in computational EAs, since they use a hand-tailored definition of the fitness function for evaluating and selecting solutions. Furthermore, many computational methods can be translated to Synthetic Biology constructs that emulate their operation, expanding the array of techniques that can be applied on a same problem and improving automation over Directed Evolution. Moreover, cell colonies of synthetic bacteria have been successfully used to build the evaluation function of an EA to evaluate the fitness of candidate solutions (Gargantilla Becerra and Lahoz-Beltra, 2020). It is in this spirit that we study Metaheuristic procedures (MH) (Glover and Kochenberger, 2006; Talbi, 2009; Sörensen, 2015), a larger class of procedures that contain EAs. Inspiration upon which these techniques are designed range from metallurgy processes (Kirkpatrick et al., 1983; van Laarhoven and Aarts, 1987; Aarts and Korst, 1988) through bird flock movement patterns (Kennedy and Eberhart, 1995; Shi, 2001; Poli et al., 2007), ant colony food foraging (Dorigo and Di Caro, 1999; Dorigo et al., 2006), and of course Darwinian evolution (Davis, 1991; Holland, 1992) (and a variant inspired on a microorganism setting (Harvey, 2009)).

In this work, we propose a general mapping, relating Synthetic Biology constructs to MH elements, such that any procedure of that class can be modeled as a synthetic circuit. This is due to MH sharing common elements and similarities that can be generalized. This alternative paradigm for designing, implementing and executing MH is developed in the context of large-scale individual systems. The original population of solutions that take part in the execution is replaced by a set of individual entities, such as cells (in this work, bacteria specifically). Therefore, large-scale parallelism is a consequence of moving toward this new paradigm. Also, the use of MH within a biological environment expands their scope by establishing a wider array of possible implementations and problems to tackle (such as Protein Design (Lippow and Tidor, 2007; Ollikainen et al., 2009; Gainza et al., 2016), or integration with Directed Evolution procedures). This association is logical, as these techniques work on a set of different elements (solutions) and apply changes to these elements to explore a large search space and eventually reach a good solution in a reasonable amount of time according to specific constraints.

MH have been long studied and possess a defined structure (Sörensen et al., 2018). One approach toward implementing AI using Synthetic Biology is shown in this article in the form of a framework that automates the mapping of MH elements to synthetic constructs. A proof-of-concept of this framework is implemented to show that automation of the process and generation of readily executable simulation files for the cell colony simulator *gro* (Jang et al., 2012; Gutiérrez et al., 2017) is feasible. It also seeks to demonstrate that by using the implementation framework, the intricacies and design of the algorithm that will be simulated need not be fully understood, since a generator automates the production of skeleton simulation files only based on specific input parameters such as conjugation rates, mutation rates, and environmental signal diffusion and degradation rates. Finally, execution speed approach is compared (in terms of generations) between simulations automatically generated by our framework implementation and standard (computational) versions of their counterparts. With this, we show that our approach enables the advantage of the intrinsic parallelism in cell colonies to explore the search space faster.

## Materials and Methods

### *gro* Simulator

*gro* (Jang et al., 2012; Gutiérrez et al., 2017) is a 2D Agent based Model (AbM) bacterial colony simulator, where the behavior of each cell is simulated individually. Bacteria grow and divide according to general parameters which are set and can be modified during simulation execution. *gro* was originally conceived as a rule-based functional language to program bacteria, but was then extended to also accept protein-based specifications to simulate the colony behavior. The simulator has a GUI which is used to visually assess the outcome of the simulation. It is especially helpful for observing spatial and temporal patterns.

After its extension, *gro* is capable of reaching 10^5^ simulated bacteria in under 10 min. Other features added were nutrient consumption, a protein-based specification language, a gene expression module to handle those dynamics automatically, and cell-cell communication in the form of a new signal module and bacterial conjugation. These improvements offer a testbed for prototyping and debugging gene circuits at an initial stage, and correcting any fundamental flaws the circuits may have. Also, the spatial and visual component of the simulator are key for identifying and determining how cell-cell communication influences the execution of the simulated gene circuits.

### MH Framework, Repositories and Hardware

To test our framework, we first generated *gro* file skeletons using Simulated Annealing (SA) and Simple Genetic Algorithm (SGA) automated generators for the specification files. These skeleton files were instances of the Boolean Satisfiability problem (SAT). Then, our team ran them on the *gro* simulator to assess their performance and demonstrate that optimal solutions could be reached using the proposed algorithms. Finally, we ran similar tests on the Cellular Automaton (CA) model, which we obtained by reusing elements generated for an SGA *gro* specification instance, and extended it into a CA instance.

We used a new version of *gro* developed by AI-UDP for the simulations. This new version can be found at https://github.com/AI-UDP/GRO63. All simulations (*gro*, C, and C++) were run in MacOS Catalina version 10.15.2 and in Windows 10. The interpreter to generate the *gro* simulation files was written in C++ and can be found at https://github.com/AI-UDP/MHInterpreter. Machines used for simulations were two MacBook Pro core i5 2.7 GHz and 2.5 GHz with 8GB RAM and a Pentium G4560 3.5 GHz with 16GB RAM.

## Results

One key aspect for using MH is to be able to represent all elements necessary for the execution of the procedure. Mainly, this involves the solution pool used in the execution, a fitness function to evaluate different solutions, and operations that carry out the exploration of new solutions. The application and design of these elements in a context of Synthetic Biology is not straightforward, as often they are dependent on the problem to solve. However, in this work, we propose a general mapping scheme to relate each of the elements which participate in an MH to a functional synthetic construct and make the association easier. The whole set of constructs is then organized and distributed over a pool of individuals (in this case, bacteria) to represent, and reproduce dynamics associated to the procedures. These constructs are designed from a general standpoint and seek to translate each of the involved components using transcriptional logic gates, intercellular communication mechanisms, and external elements such as environmental signals. It should be emphasized that the mapping presented here is a proposal and could be complemented and extended with other kinds of mechanisms, such as CRISPR (Cong et al., 2013; Xu and Qi, 2019) systems, external conditions such as temperature, nutrient consumption, or specific spatial conditions. Also, it should be stressed that our proposal heavily relies on intercellular communication, since it offers a higher computation power and also distributes it among the colony cells. The main intercell communication processes used were bacterial conjugation (Smillie et al., 2010; Goñi-Moreno et al., 2013; Cabezón et al., 2015) and Quorum Sensing (QS) (Nealson et al., 1970; Miller and Bassler, 2001; Waters and Bassler, 2005; Papenfort and Bassler, 2016).

**FIGURE 1:**
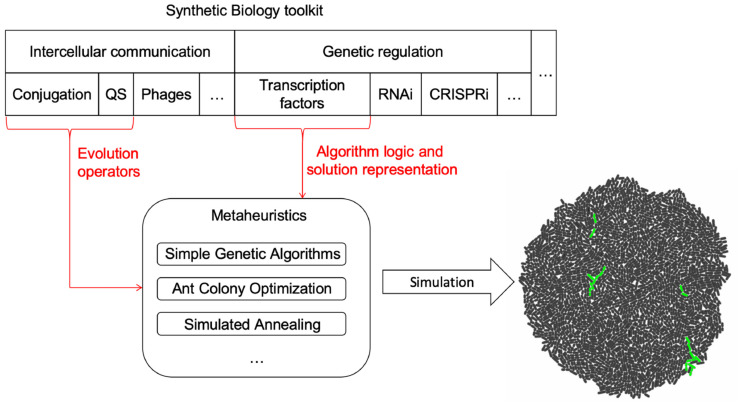
Framework components. Intercellular communication processes were chosen as the tools to implement evolution operators in MH for cell colonies. Depending on what MH is implemented, each process could play a different role. MH logic and representation are encoded using transcriptional regulation. However, other possibilities such as the use of RNA or CRISPR mediated regulation remain open to be explored as new forms of logic and representation for MH. The selected tools are then mapped to the logic of the specific MH, generating a model. Once the model and mapping have occurred, a *gro* simulation file is outputted and run to collect data on the behavior of the algorithm.

**FIGURE 2:**
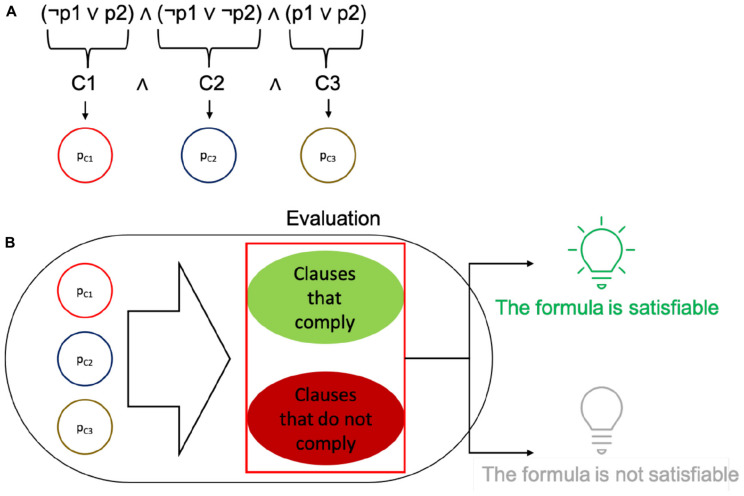
Depiction of how the SAT problem relates to the elements in cells. **(A)** Clauses are identified in a propositional formula, and each of them is encoded as a single protein within a plasmid. **(B)** The plasmids in the cell are used to evaluate the fitness of a solution (set of plasmids seeking to satisfy the formula with their truth values). The plasmids may be present or absent. According to whether the values in the plasmid contribute to the correct satisfaction of the whole formula, if it fully complies, the cell expresses GFP, else it does not glow.

**FIGURE 3:**
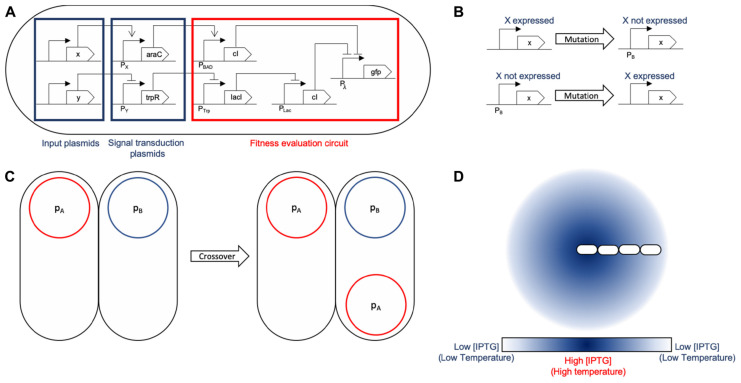
General logic mapping for SA and SGA. **(A)** General gene network mapping for the MH framework. A three-tier design is proposed: the first tier are conjugative plasmids (x and y) holding input proteins that are used by the fitness function to assess the quality of the solution. A second tier is a transition one in which the input signals are transduced into standard proteins (araC and trpR) for their evaluation. At this stage, the set of proteins which should be absent express araC to induce cI in the next tier. This is due to the fact that if any of those proteins is present, it activates cI, making the solution sub-optimal. Conversely, the set of proteins that should be present inhibit the expression of trpR, forcing the presence of all of them. If all of them are present, the following inhibition chain fails to repress GFP, evaluating correctly for those plasmids. Finally, tier three is the evaluation circuit in which the input signals are checked against their respective set (if they need to be present, absent, or it is indifferent if they are present). This part of the circuit acts works as two inverters feeding an OR gate (P_λ_): the first one (stemming from the x protein branch) evaluates for plasmids that should not be present and activates cI to block expression of GFP. The branch corresponding to the y protein evaluates for plasmids that should be present by using lacI to inhibit the expression of cI, allowing for GFP expression if all the required plasmids of that branch are present. If either condition fails, GFP will not be expressed, meaning it is not an optimal solution. However, if said evaluation is successful, it triggers GFP expression. That action may be replaced by any other such as increased conjugation rate or lower division times. **(B)** The mutation operation for SGA acts on the expression of a specific protein in the design, changing the solution to evaluate. The mutation rate parameter for SGA maps directly to the mutation rate configured in the simulation. This operation accounts for global search in terms of the solution exploration. **(C)** Crossover is a recombination operation that we mapped to bacterial conjugation. Part of a foreign solution is integrated to the current one. In our mapping, we individualized a single protein to be held by a unique plasmid, therefore mobilizing a single protein between solutions for recombination. Conjugation rate is the parameter that accounts for the SGA crossover rate parameter. **(D)** SA is largely based on a temperature decrease function: we use environmental signals (such as aTc or IPTG) as its representation. The temperature is associated to the signal concentration at a given location. The decrease is achieved by the shoving mechanical effects of the cell colony: the center of the colony experiences a greater temperature that the outer sections.

The framework is composed by three parts (a depiction of the components and their relationship can be found in Figure 1):

(1) A set of parameters that configure the execution of the instance of a MH. This set of parameters is always the same for the selected technique, despite having specific values to solve different problems. At this stage, the input parameters for the procedure are abstracted and generalized for multiple instances of the selected MH (see Table 1).
(2) A mapping/translation language to relate specific elements of the MH technique to genetic circuits. This is the fundamental idea and value of the presented work, as it provides the blueprints for automating the design of MH in Synthetic Biology (see Figures 2, 3A). How specific elements are ported to a genetic circuit will be presented further in this section.
(3) An interpreter that automates the translation of the specification of the algorithm into a skeleton of a *gro* source code, so the MH can readily be simulated and tested. The output design generation of this interpreter is based on (2) and its configuration on (1).

**TABLE 1:** Fundamental parameters for modeling MH in cell colonies.

**Algorithm parameter**	**Description**	**Algorithms**	**Biological interpretation**
*Number of proteins*	Integer that specifies the number of proteins of interest for each solution	SA, SGA	Number of plasmids expressing proteins of interest
*Protein presence*	Contribution of each protein to the fitness function (if it should be present or absent)	SA, SGA	Expected expression state of a protein (ON/OFF)
*Initial cell count*	Size of initial solution pool	SA, SGA	Initial cell colony count
*Final cell count*	Size of final solution pool (stop criterion)	SA, SGA	Final cell colony count
*Mutation rate*	Determines mutation frequency	SGA	Promoter mutation frequency
*Crossover rate*	Determines recombination rate	SGA	Conjugation rate
*Temperature decrease rate*	Establishes size and cooling rate of the temperature zone	SA	Diffusion and degradation rates of an environment signal
*Solution perturbation*	Defines how the solution is altered for exploration	SA	Conjugation rate
*Moore neighborhood size*	Establishes size of the neighborhood of a cell	CA	Diffusion and degradation rates of an intercellular signal

*Different parameters may be represented by the same biological equivalent depending on the specific algorithm to be modeled (conjugation rate, or signals for instance). All parameters marked for each MH need to be set for the execution of said simulation. This is crucial, because they control all aspects of the MH and require specification in terms of their mapped biological equivalent.*

Circuit design is done sequentially over the framework on the basis of fundamental part integration and the idea that all of the components of the MH procedure are expressed in terms of these parts. Such parts merge into a more complex circuit that evaluates the inputs, and outputs a function of these inputs. The circuits implement different elements of an MH, such as fitness function, solution representation, or evolution operators.

### Synthetic Circuit Designs for MH Simulation

Heuristics are embedded in MH through a fitness function that evaluates and guides the search for best solutions within a search space. As a base assumption in this context, we link the individual solution to the information inside a single cell. The presence or absence of a set of proteins of interest acts as the specific solution instance. This representation is inspired on the computational representation of a solution (a vector of values, which is in turn inspired on a chromosome). Therefore, depending on which proteins are present or absent, each cell represents an individual and independent solution.

Both evaluation and evolution dynamics are implemented by taking advantage of individual cell capabilities. Bacterial conjugation is used as the main backbone for evolution operations both in SA (Kirkpatrick et al., 1983; van Laarhoven and Aarts, 1987; Aarts and Korst, 1988) (as a solution mutation) and in SGA (Davis, 1991; Holland, 1992) (as recombination/crossover operation). Since solutions are represented as a set of proteins within a cell, perturbations of the set occur upon the arrival of a plasmid containing new proteins of interest into the cell. Fitness evaluation is organized in a synthetic circuit that senses the presence or absence of the proteins and performs a certain action (GFP expression in our *gro* examples) when fitness is optimal. This design decision can also be replaced by other operations such as growth rate increase/decrease or plasmid conjugation rate modification, for instance.

A summary of the proposed mappings for both SA and SGA is depicted in Figure 3.

### Framework Design and Implementation

To illustrate the capabilities and flexibility of the framework, we first present the phases involved in the parameterization, design and construction of the model along with the whole execution process associated with our implementation of the framework.

#### Parameter Collection for Model Generation

The first phase relies on user-defined parameters to guide the shaping and automated generation of the model skeleton. Specifically, two of the three techniques mentioned above (SGA and SA) are examples that can be promptly executed in cell colony simulators using our framework implementation. The third model, the CA, is shown as an example of how it would be possible to extend the functionalities and models proposed by our framework through relating to the underlying mappings and models.

The fitness function and constraints associated to each of the MH (SGA and SA) are encoded through references to proteins and their interactions: since in *gro*, proteins are the unit for directing cell behavior, in our framework, they will mainly act as the base variables. At first, the number of proteins used in the system is entered as a parameter, but also if each protein should be present, absent, or if it does not matter for describing a good solution to the problem. Concretely, the evaluation is embodied in the fitness function. Also, the initial and final number of cells in the colony for the simulation are specified as additional parameters (as a control mechanism for the simulation itself). It should also be noted that other proteins are used in the construction of the logic processes driving the algorithmic steps of each MH. Algorithm-related circuit construction process is automatically done in the next step.

These are all general parameters that are useful for specifying both SGA and SA. However, some specific parameters must also be collected in the case of each algorithm. For SGA, both mutation and crossover ratios must be provided. Mutation is implemented as an arbitrary change in the state of a protein within a cell, while crossover is simulated as a bacterial conjugation event. In the case of SA, the basic additional parameter is the temperature decrease ratio. In terms of *gro* simulations, this ratio is translated to the diffusion factor of an environmental signal (such as aTc), since the temperature can be associated to the signal concentration.

**FIGURE 4:**
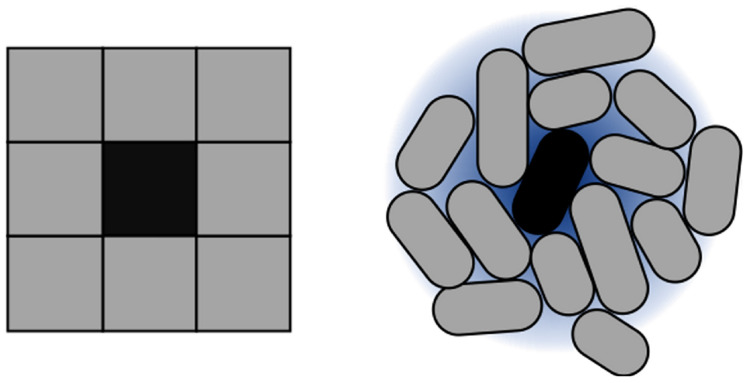
Approximation of Moore neighborhood using cells. CA execution is strongly dependent on the concept of neighborhood. 2D CA typically work on a Moore neighborhood. To reproduce this idea in the context of cell colonies, autoinducer sensing is used. The size of the neighborhood is dependent directly on the reach of the autoinducers. In terms of our simulation model, this is represented through diffusion and degradation values of the intercellular signal.

For implementing CA, they key parameter is setting intercell signaling using appropriate diffusion and degradation ratios. Such parameter configures the distance from the signaling cell to its furthest neighbor. The goal of this configuration process is to emulate the Moore neighborhood in 2D. Within these settings, rules are encoded based on the concentration of signal sensed by cells. An example is shown in Figure 4, and a summary of the parameters involved in the framework (and for CA modeling) is compiled in Table 1.

#### Translation Into the Base Model for Simulation

Once the basic parameters and elements for the MH have been chosen and put in place, a model is constructed. This model summarizes the operation rules according to the specific mapping of the different elements present in the chosen technique to simulation instructions and constructs. The models are predefined and are an extensible (although specific) representation of the algorithms. Under our framework, it is possible to capture the essence and approximate the dynamics of MH through genetic designs. Therefore, as it is possible to model a MH based on genetic circuits, it can also be simulated.

The core representation of these circuits lies in protein expression that each define the elements of the solution for all MH. Therefore, a candidate solution is linked to a set of proteins being expressed jointly within the cell (shown in Figure 3A). Intercell communication methods such as QS, or bacterial conjugation serve the purpose of providing a backbone for implementing operations on the existing solutions and obtaining new solutions. Specifically, under our representation, a set of plasmids hold the solution elements and their mobility aids in the dissemination of these elements within the colony. Since intercell communication is programmable, specific behavior regarding these operations can represent different operations for each MH.

#### Automated Generation of *gro* Skeleton Simulation File

**FIGURE 5:**
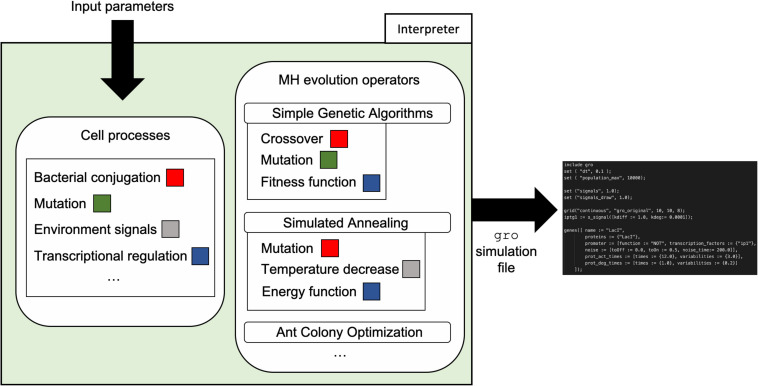
Interpreter organization and workflow. The interpreter uses mappings from cell processes to specific MH evolution operators to automatically generate a *gro* simulation skeleton file. Input parameters such as conjugation rate, mutation rate, cell population values, among others, are used for the generation of the skeleton file according to *gro* syntax.

Finally, with the model in place and informed by the input parameters, a simulation file generator constructs a *gro* simulation skeleton file. The mapping to the *gro* file takes place using the abstractions present in the simulator such as proteins, plasmids, environmental signals, etc. Our team implemented generators for two MH techniques: SA and SGA. These generators were written in C++ and require input of values for the respective parameters shown in Table 1. The generated skeleton *gro* simulation files can be directly run by the *gro* simulator, or can be modified by the final user for more specific operation. Figure 5 depicts the integration of the framework design and its implementation in the form of the interpreter.

### Simulation Implementation Examples

To test the framework implementation, we generated *gro* simulations of SGA and SA, ran the simulations, and compared their results to the ones of standard versions of the algorithms coded in C/C++. All of the simulations implement a MH for solving an adapted version of the boolean satisfiability problem (SAT) (Cook, 1971; Levin, 1973). For context, the SAT problem seeks to evaluate whether a set of truth values (true or false) assigned to propositional variables, organized in disjunctive clauses and connected through logical conjunctions, can satisfy the resulting propositional logic formula (the truth value for the whole formula is true).

In the adapted version, each boolean clause is represented by a protein under the control of a promoter as a single gene in an operon. In turn, each operon resides in a different conjugative plasmid. Hence, each clause produces a truth value that will be used to calculate the final truth value for the whole formula. Also, clauses will be arbitrarily combined within single cells, since plasmids move within the colony. In sum, each plasmid carries a boolean clause with its associated truth value.

The form of each complete solution is therefore a set of proteins within a cell, where each of them can be either present or absent. The presence of every plasmid in a cell is encoded using a bit string: if the i^th^ plasmid is present in the individual then the i^th^ bit is a 1, for the complementary case the i^th^ bit is a 0. The choice of conjugative plasmids to hold each of the proteins was made to better relate to a neighborhood space among solutions and to promote mobility of the clauses to induce combinatorial variability of the potential solutions. It should be stressed that the mentioned neighborhood is not a physical neighborhood, but a logical one for the solution set. Since a set of proteins is represented as a bit string, binary neighbors of said string can be reached through conjugation. It should be noted that, unlike the original definition of the algorithm, we did not implement plasmid loss (a possible way of finding a different solution), but only relied on plasmid mobility and aggregation. Within this binary scheme, plasmid loss would account for degradation of a protein (along with the already existing mutations). However, the intrinsic limitation of the simulator is that it works with a binary definition of proteins. These settings configure solutions to evaluate within each individual cell: a set of plasmids (bearing single proteins) present in the cell (a depiction of the setup is shown in Figure 2A).

Each solution is then evaluated by a fitness function. This function is encoded in an operon that checks for a subset of necessary proteins that should be present, a subset of detrimental proteins that should be absent, and a subset of proteins that have no effect on the fitness of the solution. The operons that implement this function are also encoded in a single plasmid. Cells that comply with the requirements of the fitness function are classified as optimal solutions. In the C/C++ versions of the simulation, an optimal solution is stored upon detection and later reported. In the *gro* simulations, optimal solutions are marked by expressing GFP, while all other bacteria are uncolored. This is done merely for simulation purposes, but GFP can be replaced with different processes such as cell death, growth rate configuration or intercell signaling, depending on the purpose of the evaluation (please see Figure 2B).

Performance of all executions was compared in terms of number of generations (iterations, in the case of the native algorithms) until finding the first instance of an optimal solution. Other performance measures are difficult to associate given differences in operation implementations and parametrization. This choice was made because a direct time comparison is not readily associated between both types of implementations, but also, because many possible interpretations of these associations are possible. Therefore, to be able to most equally compare the performance, we selected an element which is present in both types of implementations and common to most MH algorithms: generations/iterations. Thus, *gro* simulation size limits were set at 10,000 bacteria which is about 6 generations (taking into account a division time of 20 min, and 200 initial cells in the colony). This was a sufficiently number of generations, as most solutions were found within 4 generations of the start of the simulation (see Tables 2–7).

**TABLE 2:** Fitness function variation for SGA.

**Fitness function**	**First appearance of optimal solution (generation n°) – *gro***	**First appearance of optimal solution (generation n°) – C++**
11111	2.949	14.750
11000	3.072	4.862
1100	2.926	4.333
10xxx	0.628	1.000

*Data for the average number of generations before the first optimal solution appears in both versions of the simulation. The fitness function is expressed as a bit string in which a 1 in the i^*th*^ position means that the i^*th*^ plasmid must be present, while a 0 requires the plasmid to be absent. An x represents indifference for whether the plasmid is present or absent. Fitness 10xxx is found practically upon algorithm execution start. This is due to the solution 10000 being part of the initial solution pool, and matching the fitness function 10xxx. In the case of the *gro* implementation, its detection is not immediate, because it has to express the protein signaling the plasmid presence.*

**TABLE 3:** Mutation rate variation for SGA.

**Mutation rate**	**First appearance of optimal solution (generation n°) – *gro***	**First appearance of optimal solution (generation n°) – C++**
0%	4.212	17.000
1%	2.688	6.571
5%	1.540	4.833
10%	0.818	3.300

*Average number of generations it takes to find the first optimal solution with different values of mutation rate. This rate configures how often a mutation occurs per generation/iteration. In SGA, mutations represent global search operations. Therefore, a higher mutation rate speeds up finding optimal solutions, but can affect local search sequence.*

**TABLE 4:** Crossover rate variation for SGA.

**Crossover rate**	**First appearance of optimal solution (generation n°) – *gro***	**First appearance of optimal solution (generation n°) – C++**
2.5%	2.957	12.600
5%	2.621	9.600
7.5%	2.401	5.966
10%	2.129	4.966

*Average number of generations it takes to find the first optimal solution by varying the crossover rate in both SGA implementations. The number of generations required to find the optimal solution tends to decrease as the crossover rate increases. Unlike mutation, the crossover operation implements local search in the region of the search space in which the original solutions involved in recombination are located.*

**TABLE 5:** Initial population size variation for SGA.

**Population size**	**First appearance of optimal solution (generation n°) – *gro***	**First appearance of optimal solution (generation n°) – C++**
200	2.631	10.500
400	1.772	10.166
10000	0.130	9.100

*Data for the average number of generations before finding an optimal solution by varying the size of the initial population of solutions. These data show that if the initial population is larger, then less generations are needed to find an optimal individual.*

**TABLE 6:** Fitness function variation for SA.

**Fitness function**	**First appearance of optimal solution (generation n°) – *gro***	**First appearance of optimal solution (generation n°) – C**
11111	5.129	12.600
11000	1.805	11.333
1100	3.508	11.166
10xxx	0.036	13.333

*Average number of generations for each configuration to find the first instance of an optimal solution. Data show stable values for the required number of generations to find the optimal solution using the C version of the algorithm. This can be explained by the fact that the initial solution is random, and the value change of a 0 to a 1 depends on the temperature value. However, the *gro* version of the algorithm tends to behave similar to what was observed in SGA. This is, when more plasmids are required to be present in a fitness function, finding an optimal solution takes longer. Spatial location also is a factor that has a random effect over the stability: this increased randomness is two-fold since it involves specific cell location and temperature value.*

**TABLE 7:** Temperature decrease rate variation for SA.

**Degradation rate/alpha**	**First appearance of optimal solution (generation n°) – *gro***	**First appearance of optimal solution (generation n°) – C++**
0.25	2.013	1.400
0.5	1.219	6.333
0.75	3.110	6.533
0.9	2.025	8.066

*Average number of generations required to find the first instance of an optimal solution. The results show a large variability in the *gro* version of the algorithm. A possible explanation is that achieving an optimal solution depends mostly on the spatial location of the multiple solutions. Therefore, due to randomness in this location and the influence of the temperature decrease occurring outward of the colony, no clear tendency can be accurately found in these cases. However, in the C++ version of the algorithm, an increase in alpha entails an increase in the generations for finding the first optimal solution. This is due to a single solution being explored, and therefore, evolution being slowed down as the temperature decrease rate is greater.*

Finally, and as an extension to the proposed framework, we implemented a simulation in *gro* relying mainly on QS, and one in C. The goal was to map the idea of Moore neighborhood to a cell colony context (see Figure 4). To preserve a static neighborhood, we eliminated growth from the colony simulation. The implemented CA simulation was an adaptation of Conway’s Game of Life (Berlekamp et al., 1982). The synthetic circuit we propose to implement this logic is based on the idea of band detection (Basu et al., 2005; Rodríguez Regueira et al., 2019): overcrowding and under-crowding are conditions that induce grid cell death, while a mid-level crowding amount induces grid cell life. The proposed equivalence between the original Game of Life model and the *gro* simulation is that a grid cell should be mapped to a single cell in a colony.

The color code for the *gro* simulations was to use RFP for live cells and uncolored cells for “dead” ones. Cell state is determined based on the concentration of AHL at the cell location: high and low concentrations induce the cell to the “death” state, while a mid-level concentration makes the cell glow red. There are also some GFP cells, which have the task of starting the system, since there is no initial amount of AHL in the environment. These cells are placed randomly in the colony and are controlled by an environmental signal (aTc) as a start switch, and later continue their operation as normal cells.

**FIGURE 6:**
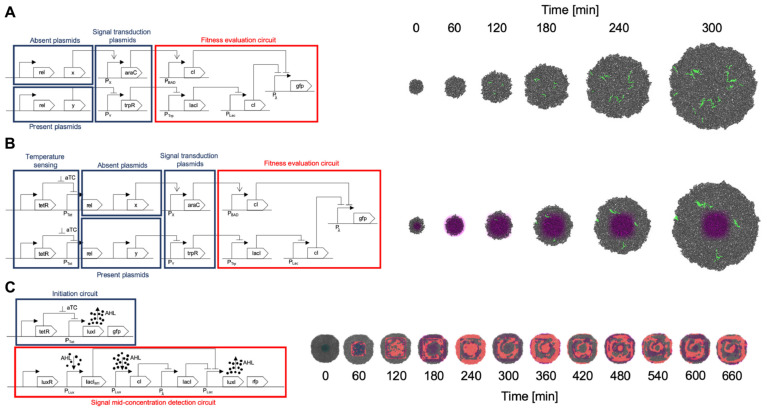
Circuit implementation and simulation. **(A)** SGA circuit implementation and simulation. The set of plasmids required to be absent for the fitness function to be optimal are organized in an OR gate manner. If any protein of this set is present, it triggers the cI repressor which in turn represses the fitness reporting (GFP for this example). The plasmids required to be present are processed through an AND gate design: if any of these proteins are missing, the trpR repressor causes the final cI repressor to act on the GFP reporting. Proteins not included in this design are omitted from the fitness evaluation, which is equivalent to a “don’t care” classification in terms of fitness values. All plasmids encoding proteins for evaluation are conjugative. Please see Figure 3A caption for details on how the input plasmids, transduction circuits, and fitness evaluation circuit were designed. **(B)** The SA implementation follows a very similar design to the one made for SGA, however, it incorporates a temperature sensing module, in which aTc concentration regulates conjugation frequency, having higher conjugation rates when aTc concentration is higher, and gradually lower ones as aTc concentration diminishes. This occurs by negatively regulating the fundamental bacterial conjugation rel protein, with the tetR repressor, and cancelling the repression in high aTc concentration zones. **(C)** A Game of Life (CA) design is implemented in two parts. It should be noted that a CA does not use a fitness function, therefore does not include this part in the implementation. One of the modules initiates the system, since no AHL signals are present in the beginning (and represent the neighborhood signaling for a “live cell”). The system starts by subjecting the cells to an aTc zone to kickstart the production of AHL (by canceling the tetR repressor). Also, these starting cells are identified by a GFP marker. Once the system is started, the first module ceases its operation and the other module, an adaptation of a band detector (Basu et al., 2005), evaluates the Game of Life in a mid-range concentration of AHL (for assessing live cells maintaining their state or dead cells coming to life). This second module is the one that keeps the CA running afterward.

A summary of the circuits implemented for all *gro* simulations is depicted in Figure 6.

#### SGA Simulation Examples

The crossover operation in these simulations is mapped to bacterial conjugation between cells. Conjugation rate is therefore associated to the crossover rate parameter of the original SGA. Mutation operation was modeled as promoter mutation leading to incorrect functioning of the circuit, and arbitrary change in protein expression. Selection is random, since arbitrary recombination occurs, and bacterial conjugation is a simulated as a stochastic process. A notable difference among both versions is that the C++ version uses a solution pool of fixed size, however, the solution pool in the *gro* version grows.

Tests were performed both on the C++ and *gro* versions by varying the fitness function to evaluate, the mutation rate, crossover rate, and population size. A baseline configuration for all simulations was: the 1,100 bit string for fitness function, 1% crossover rate, 1% mutation rate and 200 initial solutions. Using this baseline configuration, respective parameters were varied to get the results for the execution of SGA. The initial populations for all simulations were composed by 50% of empty solutions (holding no plasmids initially) and 50% holding a single plasmid, distributed equally. *gro* simulations were stopped when a population of 10,000 cells was simulated. All simulations were executed 30 times.

#### SA Simulation Examples

The C version of the SA algorithm uses the following parameters: fitness function, number of plasmids, initial temperature, the minimum temperature that marks the end of the execution of the program, and alpha (a value that denotes the decrease rate in temperature). For this instance of the model, the temperature decrease is encoded as a linear function with the alpha value being strictly lower than one. It should be noted that this version of SA works with a single solution. In contrast, the *gro* version of SA uses several solutions (each one is an individual bacterium). Therefore, an additional parameter is required: initial population size. The temperature value, in the *gro* version of the simulations, was related to an aTc global signal. This value is linked to the concentration of aTc at a given location. The temperature decrease rate (alpha) is translated to a degradation rate of the environmental signal and simulated mechanically, as cells are pushed outward and experience a lower aTc concentration (equivalent to a lower temperature).

A baseline configuration for all simulations was also set for SA: a value of 0.25 for alpha and a fitness function of 1,100. For the *gro* version, an additional default value of 200 was set for the initial population. As in SGA, initial populations for all simulations were made up of 50% of empty solutions (holding no plasmids initially) and 50% holding a single plasmid, distributed equally for the *gro* version. All simulations were executed 30 times.

#### CA Simulation Example

**FIGURE 7:**
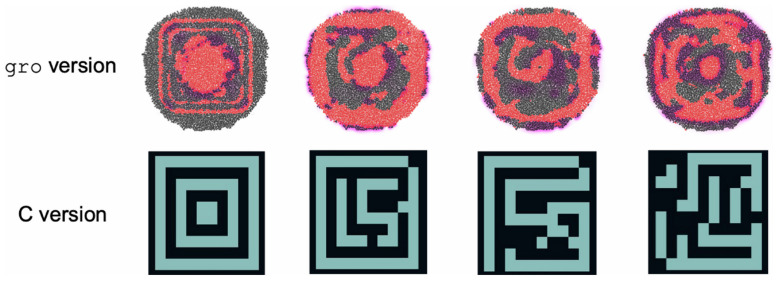
Comparison of spatial patterns achieved by Conway’s Game of Life implementations in *gro* and in C. In the *gro* implementation, red cells represent live ones, and uncolored cells represent dead ones. The magenta regions are locations at which there is a high concentration of AHL. In the C implementation, cyan cells are live ones and black cells are dead ones.

For this model, the only results that our team compared were the spatial patterns that the automaton exhibits. This comparison was made between the patterns generated by the C version and the *gro* version and is shown in Figure 7. QS was the enabling mechanism for calculating neighborhood rules in the *gro* version. A circuit based on band detection was designed to define thresholds for implementing Conway’s Game of Life.

## Discussion

We presented a new framework that proposes a mapping to associate MH to genetic circuits to be encoded in a growing cell colony. The idea behind this proposal is to bring the inspiration from evolution, used for EA (and more broadly MH), back to its origins – an evolving and growing cell colony – for assessing its viability as an implementation testbed. To the best of our knowledge, this is the first definition proposal for general MH using synthetic circuits with intercell communication as implementation backbone. The aim is to generalize how MH are defined in terms of their parameters, establish base circuits which can be extended to generically model key players in MH procedures such as fitness functions, pool of solutions, or operations. We think the presented work is a two-fold contribution as first, it eliminates the need for fully understanding all intricate mechanisms of the MH despite providing a solution for immediate use, and second, automates its design thanks to the mapping that translates all of the elements into gene circuits (that are outputted in the form of a *gro* specification file, but also set a starting point in the design of gene circuit implementation related to the MH in the wet-lab). Another advantage is a consequence of the chosen paradigm: large scale parallelism is attained by implementing MH in bacterial colonies. An interesting feature of our work is that it represents a natural way of building MH algorithms. In other words, both the final product, i.e., MH, and the approach, i.e., the principles of Synthetic Biology, are both inspired from biology. However, one constraint to which our work is subjected at this stage is that the current version of the *gro* simulator works with digital proteins. We are aware that this is a large limitation and that the power of the proposed framework would increase greatly by extending its definition to work with analog values of protein expression instead of digital values. In fact, analog values for would open an avenue for better quality and more complex evaluation schemes of solutions. Another limitation of the chosen platform is that processes involving detailed dynamics of the protein such as binding affinity, substrate specificity or protein stability are exclusively handled with a generic “mutation” operation localized at the promoter (see subsection “Parameter Collection for Model Generation”) which globally accounts for the variability in the protein operation.

Table 2 (SGA) and Table 6 (SA) in the Results section show that finding an optimal solution takes longer when a more restrictive function is sought. In the context of these simulations, the term restrictive means that more plasmids must be present simultaneously in the solution. Typically, conventional SGA and SA algorithms running in a computer use a relatively “low” number of solutions. Hence, using cell colonies, orders of magnitude more solutions can be evaluated simultaneously. This offers a larger search space exploration capability and improves speed to convergence toward good solutions (Vrajitoru, 2000; Rylander, 2002) (see Table 5). For instance, under our definition of the framework, in SGA the solution pool in a bacterial colony is constantly growing, whereas the conventional definition of SGA uses a constant sized population. This growth further improves exploration capacity since each solution evolves independently. In SA the benefits of growth are two-fold: parallel exploration of the search space, but also parallel use of the temperature values and simultaneous decrease for all solutions due to the environmental signal representing the temperature value. The comparison for population in SA was not shown, since a single solution is used in the conventional version of the algorithm.

Intrinsic processes involved in the cell and necessary for implementing our versions of MH, such as growth, gene circuit operation, or intercell communication need not be artificially imposed on growing cell colonies. However, it is their integration into the model, and the level of control which must be studied and adapted to be a suitable element within the mapping. One example involving such features may be mutations that occur in the DNA sequence: although this is a process that can be directly linked to mutations in the definition of SGA, for instance, it is also practically impossible to guarantee a mutation rate (as a parameter for SGA). It is in this spirit that we tested conditions that varied the parameter values involved in the mapping. Specifically, different biological values controlling the evolution operations of the algorithms were tested: mutation rates (Table 3), bacterial conjugation rates (Table 4), and degradation rate of an environmental signal (Table 7). The values we used for the parameters of these executions were based on realistic ones (del Campo et al., 2012; Fernandez-Lopez et al., 2014) adapted for *gro* simulation. Evolution of the solutions is accelerated as the frequency of the biological/evolution operations is increased leading to a faster convergence to an optimal solution. Mutation and crossover operations display this tendency in Tables 3, 4, and indirectly in Table 7 (since the temperature controls mutation – encoded in our SA simulations by using bacterial conjugation). In this last case, another intervening factor in our approach is evidenced: spatial location. The *gro* version of SA does not show a clear tendency in its results (although it is still faster) due to randomness of initial solution placements (related to intercell communication processes and also to the intensity of the environmental signal detected). Conventional MH lack a spatial component, while the underlying nature of cell colonies forces this component upon any synthetic procedure using intercell communication. This can be seen as an advantage, since multiple interactions occur simultaneously upon a single solution. However, it also presents a disadvantage with respect to conventional solutions in that it constrains communication to nearby cells only.

From here, a couple of possible options arise as alternatives in implementing the framework. First, a redefinition of MH within a different paradigm immersed in a biological context. Adaptation of MH to a context in which some parameters and/or elements are not fixed, controllable or can be mapped. Second, a direct and artificial mapping of the MH, forcing relationships and mappings to maintain a strict link to their original definition. Since our testbed was a simulation platform, we took a hybrid approach, leaving some of the processes, such as colony growth, to be controlled by the simulator and to be interpreted within the MH execution. This flexibility can be noted, for example, by contrasting our implementation with the definition of the original version of SA (Kirkpatrick et al., 1983; van Laarhoven and Aarts, 1987; Aarts and Korst, 1988), where there is no mention or implementation of “growth”. Our implementation also uses growth to simulate the evolution of the temperature function: due to mechanical shoving of the cells outward of the colony, it simulates temperature decrease (aTc concentration was chosen to represent the temperature measure). On the other hand, a strict link was maintained to the evolution of the solution itself and encoded in the cells as a boolean function based on plasmid and/or protein presence. This could have been modeled in a different manner and only have relied on intrinsic mutation. In sum, we propose a framework and one possible mapping for relating MH to synthetic circuits, however, other possible mappings are also valid. In spite of the proposed model being extensible, a lack of well-characterized synthetic parts may pose a problem in terms of orthogonal intercell communication (Garcillán-Barcia and de la Cruz, 2008; Grant et al., 2016; Scott and Hasty, 2016; Kylilis et al., 2018) and variety of elements to construct large synthetic circuits. Recent research has reported on possible ways for addressing this problem (Yang J. et al., 2019). Future *in vivo* implementations can be assisted by software to help select the proper parts for the design (Huynh and Tagkopoulos, 2014; Nielsen et al., 2016). The use of systems (Purnick and Weiss, 2009) (that need not be multicellular) or networks (Amos, 2014) offer a direction for tackling the lack of synthetic parts, since a large combinatorial array of circuits with varied functionalities and operation stem from their engineering and combination. Of course, multicellular distributed circuits with intercell communication should also be taken into account, as complex computation and conditions can be described (Amos, 2014; Kamm et al., 2018).

Concerning CA, QS is a key player in our implementation, driving the simulation of the model, as it relies heavily on the signal diffusion and degradation parameters. We have shown that it is possible to simulate a CA using (simulated) cell colonies (see Figures 6, 7). The patterns achieved by the *gro* simulation mimic the ones from the C simulation in their shape. Dimension discrepancies can be attributed to the difference in the calculation of the Moore neighborhood for both versions, as the *gro* version uses an AHL signal to find and sense its neighborhood, while the C version of the neighborhood is calculated directly on a grid and does not present variability. This difference makes it very difficult to faithfully reproduce an original Moore neighborhood using QS, since it is not guaranteed that the neighborhood includes a specific number of neighbors or that diffusion can be parametrized, *in vitro* or *in vivo*, to an extent that an immediate neighborhood can be detected with a very low concentration of AHL. Depending on the values related to emission and sensing, it can cause the neighborhood to extend past immediate contiguous cell neighbors, turning a cell group into what should be a single grid cell. Therefore, it has not been possible for our *gro* simulations to achieve perfect fine-grained equivalence between a bacterium and a grid cell, explaining why the patterns exhibit differences.

In consequence, the framework proposed in this work can be directed toward solving problems involving a large number of variables and in which many solutions need to be evaluated. This is based on the fact that cell colonies have very large counts and large-scale parallelism in the solution evaluations is possible. Our results suggest an intrinsic advantage of cell colony approach over conventional approaches in the reduced number of generations it takes to reach an optimal solution. We think that the number of solutions that is evaluated in parallel within this context is not something that can be achieved by a traditional computer.

In terms of applicability, protein engineering (Poluri and Gulati, 2016) is an example of a problem to which this research could be applied in that the properties or functionalities of the protein are encoded as a fitness function, establishing the selection mechanism for desired proteins to be evolved. In fact, the choice of the SAT problem for our tests was due to relation in solving subproblems of Protein Design (Lippow and Tidor, 2007; Ollikainen et al., 2009; Gainza et al., 2016). At the same time, SAT has long been researched as a problem to solve through SGA (De Jong and Spears, 1989) and SA (Spears, 1993). Being the first known NP-complete problem, it is an important one in Computer Science and has been extensively researched. We also believe that each MH implemented for cell colonies following the proposed approach, and pursuing an optimization goal, represents an intracellular specialized form of Directed Evolution. It establishes further definition and control from an algorithmic standpoint, because the general algorithmic logic and evolution steps are explicitly specified. Furthermore, this form of continuous evolution is constantly being evaluated in MH by means of a fitness function. The variability for expressing and implementing this function within the context of our framework offers improved flexibility, expressiveness and specificity in the expected solutions, acting as a complement to the original definition of Directed Evolution. By including rules defined by a MH, evolution can be controlled further and more precisely, implying a multi-level Directed Evolution technique, and maintaining compatibility with recent research involving improvements in the technique (Wu et al., 2019; Yang K. K. et al., 2019). Also, it is our opinion that the process is further made autonomous, since an additional intracellular selection machinery can be programmed and expressed in terms of synthetic circuits.

### Future Work

Integration with automated techniques for evaluating fitness functions (Gargantilla Becerra and Lahoz-Beltra, 2020) is an immediate expansion to the workflow which can lead to further automation in the definition of the algorithms to generate. Current research is also being invested into relating different AI algorithms such as Neural Networks, Reinforced Learning (Q-Learning) and other MH, such as Ant Colony Optimization, to our framework. Also, a related direction would be to further study the proposed framework by broadening the tools used to implement the underlying synthetic circuits. An idea in this direction would be to include bacteriophage infection as an intercell communication method (Ortiz and Endy, 2012) in the framework definition. The current renewed interest in AI, and its need for powerful computational resources, offers a huge opportunity for directing the potential of Synthetic Biology toward satisfying those needs and providing an alternative paradigm (and more natural, since inspiration for most MH actually comes from biology) for solving difficult problems. The goal of this ongoing and future research is to reach the definition of a global AI framework (Grozinger et al., 2019). A multi-level Directed Evolution testbed is something that also should be investigated as it is a large potential of the framework. Characterizing the power of CA in cell colonies and specifying the limits of the expressivity for these models becomes an important matter, as there are cases of CA that are Turing-complete models (Smith, 1971; Berlekamp et al., 1982). Another long-standing debt of our research group is the linkage of the *gro* simulator to accept SBOL (Madsen et al., 2019; Mısırlı et al., 2019; Baig et al., 2020; Crowther et al., 2020) specifications as input. In the context of the work presented in this paper, the association of SBOL to Agent/Individual based Model (AbM/IbM) simulators such as *gro* can go further and entail an AI toolkit within SBOL for immediate implementation of such algorithms in cell colonies.

## Data Availability Statement

The datasets presented in this study can be found in online repositories. The names of the repository/repositories and accession number(s) can be found below: https://github.com/AI-UDP/MHInterpreter.

## Author Contributions

MG and RL-B: framework design. JC: interpreter design and implementation. MG and JC: design and implementation of the *gro* versions of the algorithms. YO: design and implementation of C and C++ versions of the algorithms. MG, JC, and YO: synthetic circuit designs. YO and JC: simulation executions and data processing. MG and RL-B: wrote the manuscript. YO and MG: Supplementary Information documents. All authors have read and agreed to the published version of the manuscript.

## Conflict of Interest

The authors declare that the research was conducted in the absence of any commercial or financial relationships that could be construed as a potential conflict of interest.
